# Alterations in Mitochondrial Function in Pulmonary Vascular Diseases

**DOI:** 10.1089/ars.2024.0557

**Published:** 2025-03-07

**Authors:** Samar Farha, Kewal Asosingh, Paul M. Hassoun, John Barnard, Suzy Comhair, Andrew Reichard, Nicholas Wanner, Milena Radeva, Micheala A. Aldred, Gerald J. Beck, Erika Berman-Rosenzweig, Barry A. Borlaug, J. Emanuel Finet, Robert P. Frantz, Gabriele Grunig, Anna R. Hemnes, Nicholas Hill, Evelyn M. Horn, Christine Jellis, Jane A. Leopold, Reena Mehra, Margaret M. Park, Franz P. Rischard, W.H. Wilson Tang, Serpil C. Erzurum, the PVDOMICS Study Group

**Affiliations:** ^1^ Integrated Hospital-Care Institute, Cleveland Clinic, Cleveland, Ohio, USA.; ^2^ Lerner Research Institute, Cleveland Clinic, Ohio, USA.; ^3^ Division of Pulmonary and Critical Care Medicine, Johns Hopkins Hospital, Baltimore, Maryland, USA.; ^4^ Department of Medicine, Indiana University School of Medicine Indianapolis, Indianapolis, Indiana, USA.; ^5^ Department of Pediatrics and Medicine, Columbia University, New York, New York, USA.; ^6^ Department of Cardiovascular Medicine, Mayo Clinic, Rochester, Minnesota, USA.; ^7^ Heart Vascular and Thoracic Institute, Cleveland Clinic, Cleveland, Ohio, USA.; ^8^ Department of Environmental Medicine, New York University Grossman School of Medicine, New York, New York, USA.; ^9^ Division of Allergy, Pulmonary and Critical Care Medicine, Vanderbilt University Medical Center, Nashville, Tennessee, USA.; ^10^ Division of Pulmonary, Critical Care, and Sleep Medicine, Tufts Medical Center, Boston, Massachusetts, USA.; ^11^ Division of Cardiology, Weill Cornell Medical Center, New York, New York, USA.; ^12^ Division of Cardiovascular Medicine, Brigham and Women’s Hospital, Harvard Medical School, Boston, Massachusetts, USA.; ^13^ Division of Pulmonary, Critical Care and Sleep Medicine, University of Washington, Seattle, Washington, USA.; ^14^ Division of Pulmonary, Allergy, Critical Care and Sleep Medicine, University of Arizona, Tucson, Arizona, USA.

**Keywords:** pulmonary hypertension, mitochondria, arginine metabolism, transmembrane potential, superoxide production

## Abstract

**Aims:**

Alterations of mitochondrial bioenergetics and arginine metabolism are universally present and mechanistically linked to pulmonary arterial hypertension (PAH), but there is little knowledge of arginine metabolism and mitochondrial functions across the different pulmonary hypertension (PH) groups. We hypothesize that abnormalities in mitochondrial functions are present across all PH groups and associated with clinical phenotypes. We test the hypothesis in PH patients and healthy controls from the Pulmonary Vascular Disease Phenomics Program cohort, who had comprehensive clinical phenotyping and follow-up for at least 4 years for death or transplant status. Mitochondrial transmembrane potential, superoxide production, and mass were measured by flow cytometry in fresh platelets. Metabolomics analysis was performed on plasma samples. Global arginine bioavailability was calculated as the ratio of arginine/(ornithine+citrulline).

**Results:**

Global arginine bioavailability is consistently lower than controls in all PH groups. Although the mitochondrial mass is similar across all PH groups and controls, superoxide production and transmembrane potential vary across groups. Mitochondrial superoxide is higher in group 1 PAH and lowest in group 3 compared with other groups, while transmembrane potential is lower in group 1 PAH than controls or group 3. The alterations in mitochondrial functions of group 1 PAH are associated with changes in fatty acid metabolism. Mitochondrial transmembrane potential in group 1 PAH is associated with transplant-free survival.

**Conclusion:**

While alterations in mitochondrial function are found in all PH groups, group 1 PAH has a unique mitochondrial phenotype with greater superoxide and lower transmembrane potential linked to fatty acid metabolism, and clinically to survival. *Antioxid. Redox Signal.* 42, 361–377.

**Innovation**

There is a need to expand our understanding of metabolic abnormalities and mitochondrial dysfunction across the spectrum of pulmonary vascular diseases. This is the first study measuring mitochondrial function and arginine bioavailability in a large cohort of well-phenotyped patients with PH and across all five groups in comparison with healthy controls. Our findings highlight the decreased arginine bioavailability across all PH groups and heterogeneity in platelet mitochondrial function among the different groups, and reveal shifts in fuel use that link to disease types and severity.

## Introduction

Pulmonary arterial hypertension (PAH) is a progressive and often lethal disorder disproportionately afflicting women and is a subclass of a broader group of pulmonary vascular diseases. Pulmonary vascular diseases are classified by clinical conditions associated with pulmonary hypertension (PH) based on similar pathophysiological mechanisms, clinical presentation, hemodynamics, and therapeutic management into five groups. Group 1 (G1) PAH includes idiopathic, heritable, drug- and toxin-induced, associated forms, PAH with features of venous/capillary involvement, and persistent PH of the newborn. G2 PH is secondary to the left heart diseases, G3 is associated with lung diseases or hypoxia, G4 is due to chronic pulmonary artery obstruction, and G5 includes diseases with multifactorial or unclear mechanisms (Humbert et al., 2022; Simonneau et al., 2013).

Multiple lines of evidence from preclinical models, human cells derived from PAH lungs, and studies of patients identify abnormalities of metabolic pathways in the molecular pathogenesis of PAH, including abnormalities in mitochondrial numbers, mitochondrial oxidative phosphorylation, decreased electron transport chain (ETC) function in vascular cells from PAH lungs, and shift to greater glycolysis and glucose uptake in hearts, lungs, and skeletal muscles in PAH patients (Bonnet et al., 2006; Egnatchik et al., 2017; Malenfant et al., 2015; Pak et al., 2013; Xu et al., 2021; Xu et al., 2007). In parallel to bioenergetic changes, PAH is mechanistically linked to abnormalities of arginine metabolism. Arginine is the substrate for endothelial nitric oxide (NO) synthase, which converts arginine to NO and citrulline. Loss of NO production in PAH is associated with greater mitochondrial arginine metabolism *via* arginase, which converts arginine to ornithine (Ghosh et al., 2016; Kaneko et al., 1998; Klinger et al., 2013; Machado et al., 2004; Ozkan et al., 2001; Tonelli et al., 2013; Xu et al., 2004; Xu et al., 2007). Thus, alterations of arginine/NO metabolism are biochemically linked to abnormalities of mitochondrial bioenergetics (Nisoli and Carruba, 2006).

With early reports of metabolic interventions for the treatment of PH showing encouraging results (Archer et al., 2010; Fang et al., 2012; Farha et al., 2017; Michelakis et al., 2017; Paddenberg et al., 2007; Piao et al., 2013; Piao et al., 2010; Seyfarth et al., 2013; Sharma et al., 2013; Sharp et al., 2014), targeting mitochondrial pathways offers a promising approach to developing new therapeutic options. However, in multifactorial diseases such as PH, mitochondrial function may vary from patient to patient, and over time, and may be affected by various factors, including therapies, underscoring the need for a precision approach in applying metabolic interventions. There is a need to quantify mitochondrial function, a task limited by the lack of ready availability of mitochondria from affected tissues. Recent work suggests that platelets can serve as a valid measure of mitochondrial function in diseases (Ben-Shachar et al., 2007; Guo et al., 2009; Parker et al., 1990a, 1990b; Parker et al., 1989; Rezania et al., 2017; Sangiorgi et al., 1994; Shi et al., 2008; Zharikov and Shiva, 2013). Platelets may be a particularly important site to study mitochondria in PAH. *In situ* thrombosis and abnormal platelet aggregation are hallmarks of PAH, and platelets have been implicated in PAH pathogenesis through the release of proinflammatory, angiogenic, and prothrombotic mediators (Aytekin et al., 2012; Herve et al., 2001; Lannan et al., 2014).

Despite substantial knowledge on alterations in mitochondrial bioenergetics and arginine–NO metabolism in G1 PAH, there is still a need to quantify mitochondrial function, which is limited by access to mitochondria from affected tissues. Furthermore, there is a gap in our understanding of these pathways in other PH groups. In this study, we hypothesize that (1) abnormalities in mitochondrial function are present in PH and are associated with clinical groups and/or phenotypes and (2) platelets can be used to assess the metabolic shift and mitochondrial dysfunction of the pulmonary vascular bed. We explore metabolic pathways associated with mitochondrial activity to uncover mechanistic connections between mitochondrial function and PH. The findings are summarized in the Graphical Abstract.

## Results

### Study population

The study population of the Pulmonary Vascular Disease Phenomics (PVDOMICS) cohort has been previously described (Hemnes et al., 2022). The subgroup for this analysis consists of 70 healthy controls and 461 patients with PH. Demographics are outlined in Table 1. While there is no difference in race distribution, there is a significant difference in age and sex distribution between healthy controls and PH participants. Healthy controls are younger and have a higher proportion of women compared with PH participants (Table 1).

**Table 1.** Demographics of Study Cohort

	Controls	PH	*p* Value
	*N* = 70	*N* = 461	
Age (years)	47 ± 14	59 ± 15	<0.001
Race, *n* (%)			0.2
Caucasian	59 (84%)	354 (77%)	
African American	9 (13%)	65 (14%)	
Others	2 (3%)	42 (9%)	
Sex, *n* (%)			0.05
Female	52 (74%)	286 (62%)	
Male	18 (26%)	175 (38%)	
Pulmonary hypertension groups, *n* (%)			
Group 1		202 (44%)	
Group 2		90 (19%)	
Group 3		109 (24%)	
Group 4		41 (9%)	
Group 5		19 (4%)	
PAH-specific therapies, *n* (%)			
Phosphodiesterase type 5 inhibitors/stimulator of soluble guanylate cyclase		209 (45%)	
Endothelin receptor antagonist		134 (29%)	
Prostacyclin/prostacyclin receptor agonist		95 (21%)	
Temperature (Fahrenheit)	98.0 ± 0.6	97.9 ± 0.6	0.1
Pulse (beats/min)	71 ± 11	77 ± 14	<0.001
O_2_ saturation (%)		94 ± 4	<0.001
RVSP (mmHg)	24 ± 5	60 ± 23	<0.001
Hemodynamic variables			
Right atrial pressure (mmHg)		7 [4, 11]	
Mean pulmonary arterial pressure (mmHg)		38 [30, 49]	
Pulmonary capillary wedge pressure (mmHg)		12 [8, 16]	
Cardiac output (l/min)		5.2 ± 1.8	
Pulmonary vascular resistance (Wood units)		4.8 [3.1, 7.7]	
6-Minute walk distance (m)	529 ± 101	337 ± 140	<0.001
NT-proBNP (pg/mL)	48 [23, 74]	331 [112, 1423]	<0.001
WHO functional class (I/II/III/IV)		33/150/235/25	

Statistics presented as mean ± SD, median [P25, P75], N (column %).

PH, pulmonary hypertension; PAH, pulmonary arterial hypertension; O_2_, oxygen; RVSP, right ventricular systolic pressure; NT-proBNP, N-terminal pro-brain natriuretic protein; WHO, World Health Organization.

### Effect of sex, race, and age on mitochondrial mass and function

**FIG. 1.**
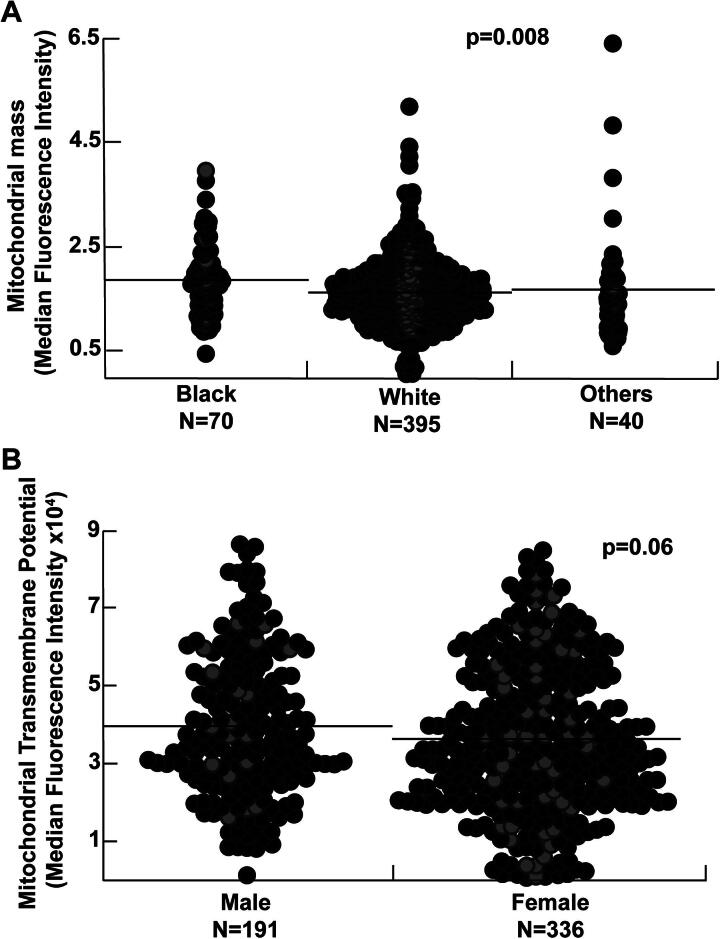
**Effect of sex and race on mitochondrial assays.** **(A)** Mitochondrial mass was significantly higher in Black participants. Mitochondrial transmembrane potential and superoxide production were not affected by race. **(B)** Male participants tended to have higher mitochondrial transmembrane potential. Mass and superoxide production were not affected by sex. Individual data points shown. Black line represents the mean. Gray circles represent controls, and black circles pulmonary hypertension (PH). Wilcoxon *p* value used.

**FIG. 2.**
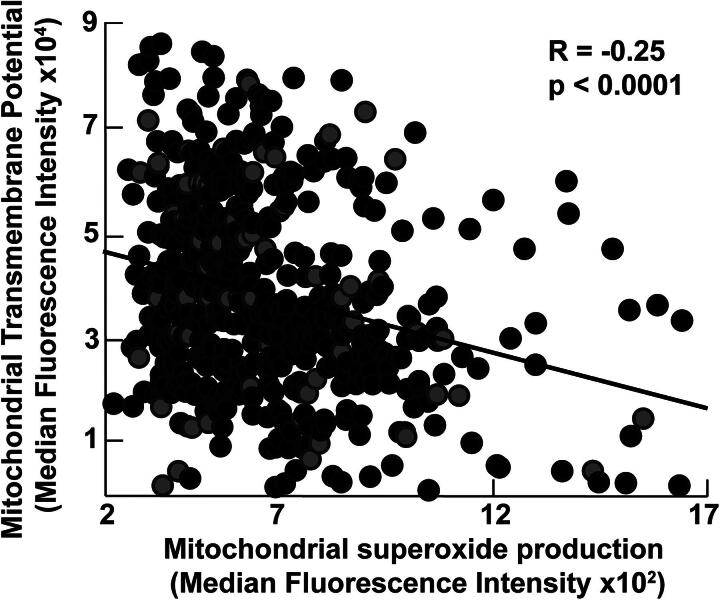
**Mitochondrial transmembrane potential is inversely related to superoxide production.** In our overall cohort, there is an inverse correlation between mitochondrial transmembrane potential and superoxide production. Pearson’s correlation used with line of best fit shown for visualization.

Sex, race differences, and aging-related changes in mitochondrial biogenesis and bioenergetics are described in health and disease states. We thus explored the effect of sex, age, and race in our cohort and found that the mitochondrial mass is higher in Black participants (*p* = 0.008) and inversely related to age (*R* = −0.1, *p* = 0.03) (Fig. 1). Mitochondrial transmembrane potential and superoxide production do not correlate with race and age. Moreover, mitochondrial mass and superoxide production are not affected by sex. Transmembrane potential tends to be lower in women (*p* = 0.06) (Fig. 1). All analyses of mitochondrial assays are thus adjusted for age, sex, and race. Among mitochondrial measures, the mitochondrial mass correlates weakly with mitochondrial superoxide production (*R* = 0.15, *p* < 0.001). There is no correlation between mitochondrial mass and transmembrane potential in the study cohort (*p* = 0.2). On the contrary, transmembrane potential is inversely related to superoxide production (*R* = −0.25, *p* < 0.001) (Fig. 2).

**FIG. 3.**
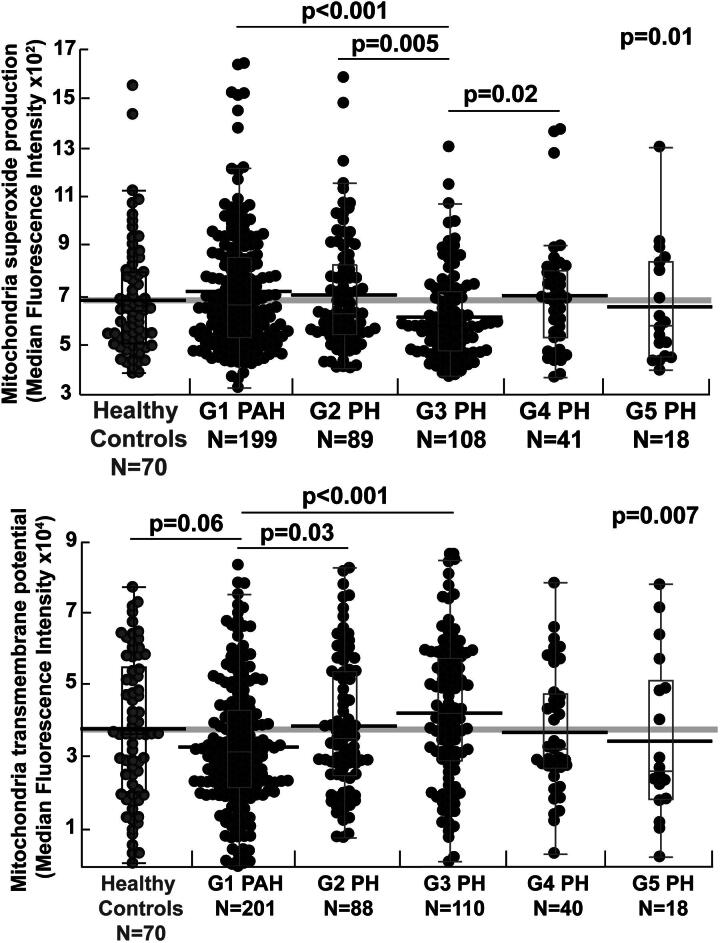
**Group1 (G1) pulmonary arterial hypertension (PAH) has lower mitochondrial transmembrane potential and higher superoxide production.** Mitochondrial transmembrane potential and superoxide production varied across PH groups. G1 PAH had significantly lower transmembrane potential compared with G2 and 3 PH and a tendency for lower transmembrane potential compared with healthy controls. G3 PH had lower superoxide production compared with G1, 2, and 4 PH. Gray circles represent controls and black circles PH. Box plots show median and 25%−75% quartiles. Whiskers mark the maximum and minimum values excluding outliers. The gray thick line represents the mean for healthy controls. Black thick lines represent the mean of each corresponding group. Wilcoxon *p* values for overall comparison and pairwise comparisons are shown. Bonferroni-adjusted significance level required *p* < 0.005.

### Alterations in mitochondrial transmembrane potential and superoxide production

There are no significant differences in mitochondrial mass or function between healthy controls and all patients with PH (all *p* > 0.1). While mitochondrial mass does not differ by PH groups, mitochondrial function is significantly different across the PH groups. G3 PH has the lowest superoxide production, significantly lower than G1 and G2 PH. G1 PAH tends to have a lower mitochondrial transmembrane potential compared with healthy controls and G2 PH and significantly lower transmembrane potential compared with G3 PH (Table 2) (Fig. 3).

**FIG. 4.**
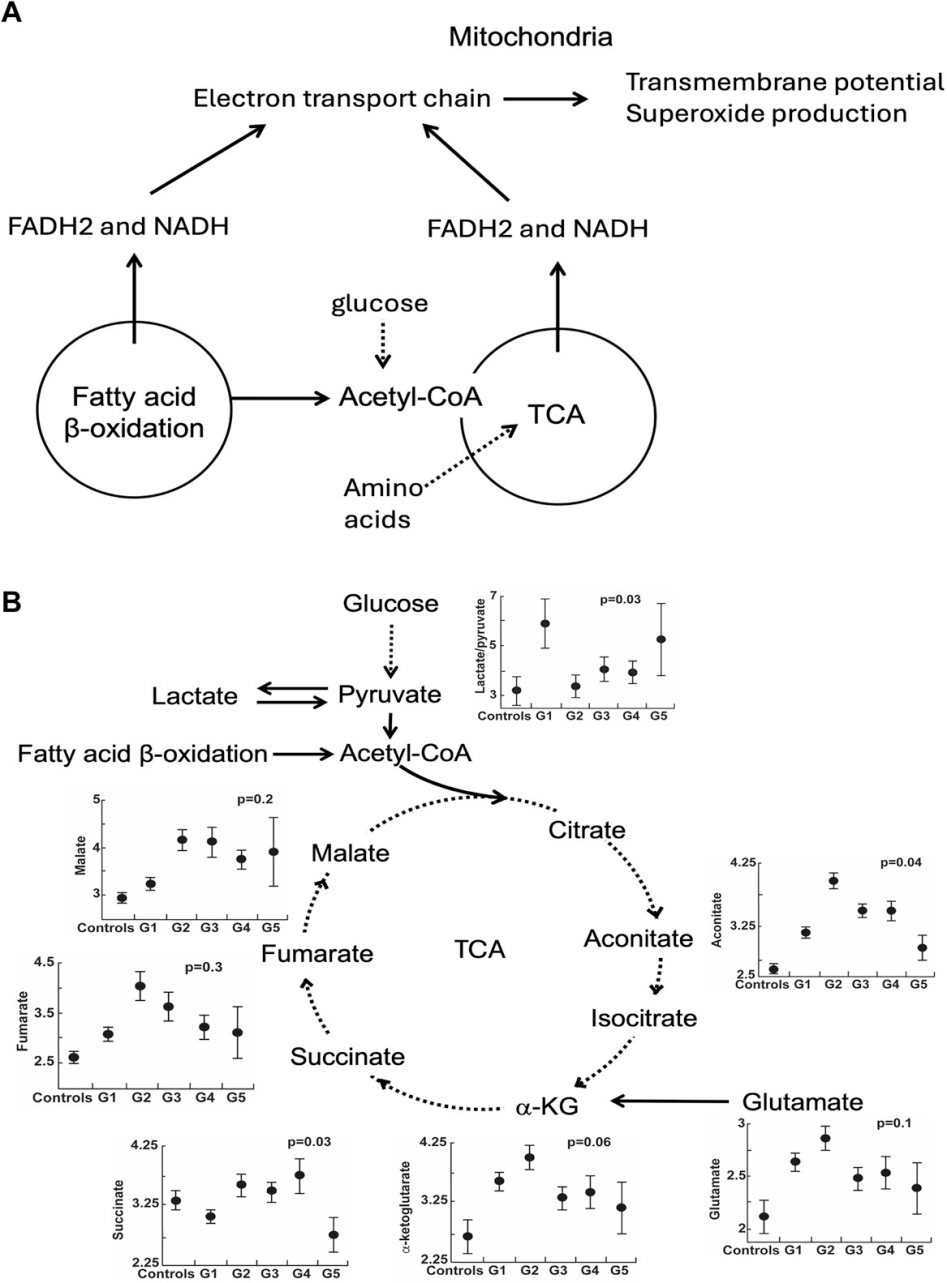
**Mitochondrial electron transport chain (ETC) and the tricarboxylic acid (TCA) cycle.** **(A)** ETC, a series of protein complexes in the inner mitochondrial membrane, picks electrons from reduced electron carriers (NADH/FADH2) produced by the TCA cycle and fatty acid β-oxidation creating an electrochemical gradient that leads to the production of ATP. Superoxide is produced during the process. Fatty acid β-oxidation transfers electrons to the ETC directly through complex III or by generating acetyl-coA that goes into the TCA cycle. **(B)** TCA cycle metabolites are different among PH groups and controls with an elevated lactate-to-pyruvate ratio noted in G1 PAH. Data shown as mean with Standard Error of the Mean (SEM). *p* Value adjusted for age, race, sex, and estimated glomerular filtration rate (eGFR).

**Table 2.** Mitochondrial Assays and Arginine Pathway (Targeted) Metabolites Across Pulmonary Hypertension Groups and Controls

	Controls	G1 PAH	G2 PH	G3 PH	G4 PH	G5 PH	*p* Value
Mitochondrial mass (MFI)	1.6 [1.2, 1.9]	1.6 [1.2, 2.0]	1.6 [1.3, 1.9]	1.5 [1.2, 1.9]	1.7 [1.3, 2.0]	1.8 [1.3, 2.2]	0.9
Mitochondrial superoxide production (MFI ×10^2^)	6.3 [5.0, 8.0]	6.6* [5.3, 8.5]	6.2 [5.4, 8.2]	5.7* [4.7, 7.2]	6.9 [5.3,8.0]	5.8 [4.5, 8.4]	0.01
Mitochondrial transmembrane potential (MFI ×10^4^)	3.7 [2.1, 5.6]	3.2* [2.2, 4.4]	3.6 [2.6, 5.5]	4.2* [2.9, 5.8]	3.3 [2.8, 4.8]	2.7 [1.9, 5.2]	0.007
Arginine (µM)	104.1 [88.3, 123.6]	99.6 [82.0,120.6]	94.3 [81.2, 116.1]	98.7 [77.7, 124.0]	109.5 [87.8, 125.8]	106.2 [73.5,129.7]	0.4
Ornithine (µM)	66.8 [55.8, 81.7]	73.6* [54.9, 89.0]	85.8*^,†,^*^#^* [70.5, 145.6]	71.8† [60.5, 88.2]	76.5 [63.1, 90.5]	77.3 [58.9, 82.4]	0.04
Citrulline (µM)	34.5 [27.1, 42.7]	39.1*^,#^ [32.2, 47.5]	49.4*^,†,^*^#^* [36.3, 65.0]	37.6† [30.5, 46.8]	41.1*^#^* [34.2, 52.2]	40.0 [31.5, 57.9]	<0.001
GABR	1.08 [0.91, 1.26]	0.94*^,^*^#^.* [0.73, 1.14]	0.76*^,^†^,^*^#^* [0.54, 0.93]	0.88†^,^*^#^* [0.71, 1.11]	0.85*^#^* [0.67, 1.14]	0.92 [0.73, 1.13]	0.009
Urinary nitrate/creatinine (µmol/µmol)	0.07 [0.04, 0.09]	0.07* [0.04, 0.09]	0.04* [0.03, 0.08]	0.06 [0.04, 0.09]	0.05 [0.03, 0.07]	0.04 [0.03, 0.08]	0.4

Statistics presented as median [P25, P75], Kruskal–Wallis *p* value adjusted for age, sex, and race. Significant pairwise analyses between PH groups are marked with *, †. PH groups that are significantly different from controls are marked with #. Bonferroni-adjusted significance level <0.003 for multiple comparisons.

GABR, global arginine bioavailability ratio; MFI, mean fluorescence intensity.

PH therapies and management vary based on PH groups with most PH-specific therapies used in G1 PAH and G4 PH. In the PH cohort, 82% of G1 PAH patients are on PH therapies compared with 24% of G2 PH, 35% of G3 PH, 37% of G4 PH, and 53% of G5 PH patients. Mitochondrial transmembrane potential is lower in patients on PH therapies (*p* < 0.001), whereas superoxide production is not different between the two groups (*p* = 0.5). Likewise, when comparing prevalent cases with incident cases, mitochondrial transmembrane potential is lower and superoxide production higher in prevalent cases compared with incident (both *p* < 0.01). Mitochondrial transmembrane potential is inversely associated with years since PH diagnosis (*R* = −0.15, *p* = 0.002). Propensity score analysis using sex, age, race, and PH medications as confounders reveals a significant difference in transmembrane potential and superoxide production among all PH groups (both *p* ≤ 0.01). Superoxide production is significantly higher in G1 compared with G3 PH using Bonferroni-adjusted significance level <0.005 for pairwise comparison (Supplementary Table S1).

Another important clinical aspect to PH grouping is that patients often have a mixed etiology of their disease, that is, they fit more than one of the current PH classification groups. In this cohort, 39% have overlap of PH classification (Hemnes et al., 2022). To adjust for overlap in classifications, analyses are also performed with exclusion of patients with mixed groups (*N* = 183). The differences in mitochondrial transmembrane potential and higher superoxide production between G1and G3 PH remain statistically significant (both *p* < 0.001) after excluding patients with mixed PH etiologies.

### Arginine–NO pathway

Arginine–NO pathway abnormalities are well described in preclinical models of PH and in G1 PAH, and abnormalities are associated with abnormal bioenergetics (Afolayan et al., 2016; Aytekin et al., 2012; Farha et al., 2021; Ghosh et al., 2016; Kao et al., 2015; Xu et al., 2004), but little is known about this pathway in other PH groups. Targeted metabolomics of arginine pathway metabolites across PH groups and controls shows that arginine is not different among PH and controls (arginine [µM]: controls 104.3 [88.3, 123.6], PH 98.4 [80.9, 120.7]; *p* = 0.2). However, citrulline and ornithine levels are significantly higher in PH compared with controls (citrulline [µM]: controls 34.5 [27.1, 42.7], PH 39.7 [32.3, 51.2]; *p* < 0.001 and ornithine [µM]: controls 66.8 [55.8, 81.7], PH 75.7 [59.0, 93.0]; *p* = 0.02). Thus, similar to prior reports of G1 PAH (Kao et al., 2015; Morris et al., 2005; Simpson et al., 2023; Xu et al., 2021), the global arginine bioavailability ratio (GABR) is significantly lower in PH compared with controls (GABR: controls 1.08 [0.9, 1.3], PH 0.88 [0.69, 1.10]; *p* < 0.001). There is no significant difference between the groups in urinary nitrate, a stable salt of NO excreted in urine (*p* = 0.1) (Xu et al., 2007).

Arginine is also not different among the various PH groups (*p* = 0.5). Citrulline and ornithine vary significantly among groups with higher levels in G2 PH (Table 2). GABR varies among PH groups with lower levels in G2 PH (Table 2). Results remain significant after using propensity score to adjust for PH medication use (Supplementary Table S1). Urinary nitrate is higher in G1 PAH compared with G2 PH (Table 2); however, this is not significant after adjusting for PH medications using propensity score analysis. These analyses show a previously unknown heterogeneity of the arginine–NO pathway among PH groups, and provide insight to the pathophysiology of the different cardiovascular phenotypes.

### Mitochondrial function and bioenergetics: the tricarboxylic acid cycle

Both tricarboxylic acid (TCA) cycle and fatty acid β-oxidation use fuels in reactions to provide Nicotinamide Adenine Dinucleotide (NADH) and Flavin Adenine Dinucleotide (FADH2) for the ETC in the inner mitochondrial membrane to pump protons and generate a transmembrane potential that is used to produce energy, heat, and reactive oxygen species (ROS). In this study, abnormalities of metabolism are related to mitochondrial transmembrane potential and superoxide production using nontargeted metabolomic data. The differences in TCA metabolites among PH groups and controls are shown in Figure 4 and Table 3. TCA metabolites dependent on mitochondrial complex 1 NADH dehydrogenase activity differ across PH groups and controls (Fig. 4). There is a marked increase of lactate-to-pyruvate ratio in G1 PAH, which identifies a shift to glycolysis and less oxidative metabolism *via* TCA cycle, as described in prior studies (Fessel et al., 2012; Fijalkowska et al., 2010; Tuder et al., 2012; Xu et al., 2021; Xu et al., 2007).

**FIG. 5.**
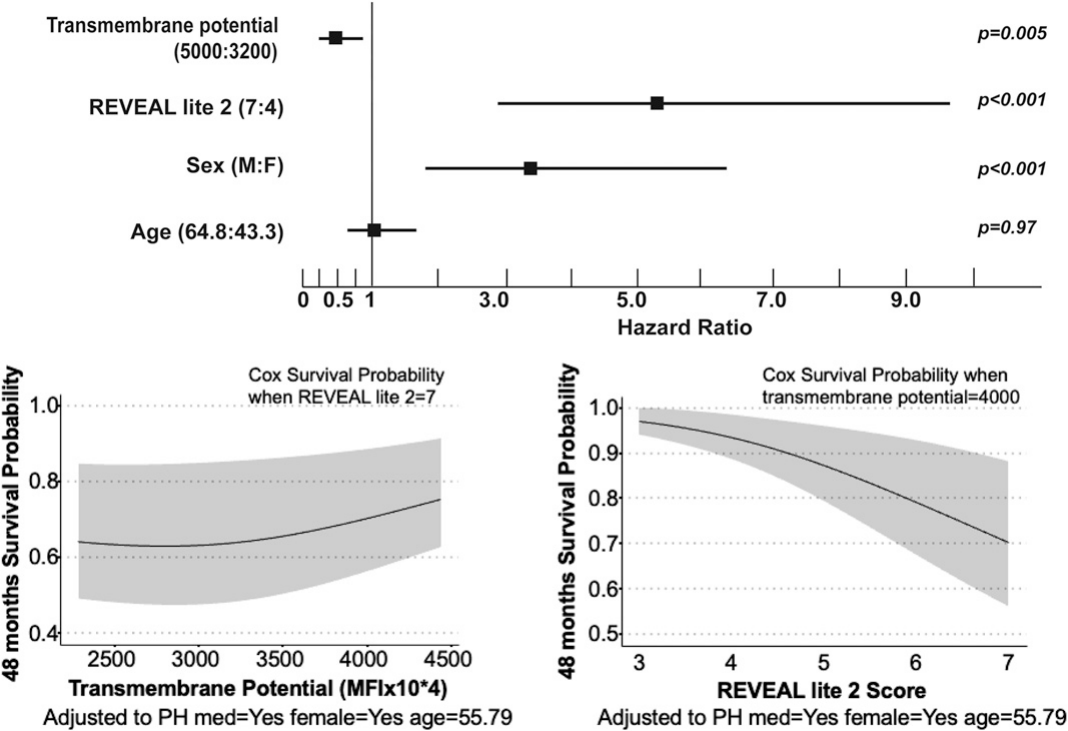
**Mitochondrial transmembrane potential is a predictor of survival in G1 PAH.** We performed Cox proportional hazard survival analysis to predict clinical outcomes using predictors age, sex, REVEAL LITE 2, and transmembrane potential. Only in G1 PAH, transmembrane potential was a predictor of survival (hazard ratio = 0.45 [0.25–0.79], *p* = 0.009). Forty-eight months of survival plots are shown for transmembrane potential and REVEAL LITE 2 score. In contrast to REVEAL LITE 2, which demonstrates a robust predictive value for survival across its entire range, transmembrane potential exhibits a nonlinear association. Specifically, at lower values, the survival curve remains relatively flat, while at higher transmembrane potential values (>3000–3500 × 10^4^), there is a significant association with improved survival.

**Table 3.** Tricarboxylic Acid Metabolites (Untargeted) Across Pulmonary Hypertension Groups and Controls

	Controls	G1 PAH	G2 PH	G3 PH	G4 PH	G5 PH	FDR
Lactate	3.53 [2.81, 4.48]	3.05 [2.33, 3.66]	3.23 [2.71, 4.05]	3.39 [2.63, 4.39]	3.36 [2.66, 3.77]	3.07 [2.50, 4.39]	0.01
Pyruvate	1.66 [1.04, 2.54]	1.00 [0.51, 1.83]	1.44 [0.74, 2.92]	1.11 [0.64, 2.13]	1.04 [0.63, 1.66]	1.18 [0.33, 2.70]	0.05
Succinate	3.23 [2.71, 3.89]	2.90 [2.38, 3.71]	3.56 [2.96, 4.46]	3.33 [2.62, 4.28]	3.43 [2.81, 4.60]	2.68 [2.42, 3.37]	0.03
Aconitate	2.49 [2.22, 3.93]	2.97 [2.40, 3.72]	3.90 [3.27, 4.42]	3.33 [2.75, 4.25]	3.46 [2.91, 4.05]	2.97 [2.28, 3.57]	0.04
2-Methylcitrate/homocitrate	0.85 [0.04, 1.50]	1.21 [0.14, 1.98]	1.59 [1.12, 2.74]	1.31 [0.08, 1.88]	1.09 [0.38, 1.90]	1.58 [1.02, 2.57]	0.01
Glutamate	1.88 [1.13, 2.61]	2.42 [1.81, 3.13]	2.79 [2.16, 3.40]	2.26 [1.71, 3.15]	2.45 [1.76, 3.02]	2.30 [1.61, 2.59]	0.1
Malate	2.83 [2.45, 3.23]	2.85 [2.28, 3.81]	3.77 [2.93, 4.66]	3.26 [2.78, 4.23]	3.83 [2.95, 4.22]	2.95 [2.52, 3.70]	0.2
Fumarate	2.45 [1.98, 2.98]	2.68 [2.04, 3.63]	3.52 [2.75, 4.54]	2.82 [2.23, 3.76]	3.10 [2.41, 3.75]	2.43 [2.05, 3.36]	0.3
α-Ketoglutarate	2.36 [2.02, 3.04]	2.56 [2.05, 3.56]	3.58 [2.92, 4.57]	2.95 [2.47, 4.97]	2.88 [2.46, 3.86]	2.86 [1.96, 3.07]	0.06

Statistics presented as median [P25, P75], FDR across all groups adjusted for age, sex, race, eGFR.

FDR, false discovery rate; eGFR, estimated glomerular filtration rate.

### Metabolic pathways associated with mitochondrial function

To further explore metabolic pathways associated with the change in mitochondrial transmembrane potential and superoxide production, untargeted metabolomics of circulating metabolites is correlated to mitochondrial measures. A total of 22 known metabolites are associated with transmembrane potential in PH at false discovery rate (FDR) <0.05. Most of these metabolites are in the fatty acid metabolic pathways. The interaction effect of PH group on the metabolite–transmembrane potential relationships is significant in 16 metabolites when comparing G1 with G2 or G3 (FDR <0.05). These metabolites are also primarily in the fatty acid pathways. Pathways associated with transmembrane potential in G1 compared with G2 and G3 include fatty acid acyl carnitine metabolism, fatty acid dicarboxylate and monohydrate, as well as androgenic steroids and corticosteroids (FDR <0.05). Acyl carnitine pathways, which are important in fatty acid oxidation, have the highest fraction of differential metabolites (Table 4). The top two metabolites in the pathway that interact differently with transmembrane potential in G1 *versus* G2–3 are octadecenedioylcarnitine (C18:1-DC) (FDR = 0.005) and suberoylcarnitine (C8-DC) (FDR = 0.01). In addition, fatty acid (acyl carnitine, dicarboxylate) metabolism and dicarboxylate metabolites are significantly different among PH groups and controls (Table 5).

**Table 4.** Fatty Acid Metabolism Pathways and Metabolites Associated Differentially with Mitochondrial Transmembrane Potential in Group1 *Versus* Groups 2–3 Pulmonary Hypertension

Pathway	Metabolite	TDP	FDR
Fatty acid (acyl carnitine, dicarboxylate) metabolism		0.4	0.009
	Octadecenedioylcarnitine (C18:1-DC)		0.005
	Suberoylcarnitine (C8-DC)		0.02
Fatty acid, dicarboxylate		0.16	0.01
	Hexadecenedioate (C16:1-DC)		0.005
	Hexadecanedioate (C16)		0.04
	Octadecenedioate (C18:1-DC)		0.04
	Tetradecanedioate (C14)		0.05
	Octadecanedioate (C18)		0.07
	3-Hydroxydodecanedioate		0.07
	Tetradecadienedioate (C14:2-DC)		0.07
	3-Hydroxyadipate		0.09
Fatty acid, monohydroxy		0.17	0.04
	3-Hydroxyhexanoate		0.01
	3-Hydroxysebacate		0.01
	16-Hydroxypalmitate		0.03

TDP represents the proportion of truly different metabolites in the pathway. FDR represents the comparison of the association of pathway or metabolite with transmembrane potential in G1 compared with G2–3 PH. Model adjusted for age, race, sex, eGFR, and PH medications.

TDP, true discovery proportion; G1, group 1; G2–3, groups 2–3.

**Table 5.** Differences in Fatty Acid Metabolites (Acyl Carnitine, Dicarboxylate Metabolism, and Dicarboxylate Pathway) Across Pulmonary Hypertension Groups and Controls

Pathway	Metabolite	Controls	G1 PAH	G2 PH	G3 PH	G4 PH	G5 PH	FDR
Fatty acid (acyl carnitine, dicarboxylate) metabolism								
	Octadecenedioylcarnitine (C18:1-DC)	0.9 [0.6, 1.4]	1.7 [1.1, 2.6]	2.3 [1.3, 2.3]	2.0 [1.2, 3.0]	2.1 [13, 2.8]	1.6 [1.0, 2.1]	<0.001
	Suberoylcarnitine (C8-DC)	0.5 [0.1, 0.7]	1.4 [0.7, 2.5]	1.9 [1.1, 3.1]	1.3 [0.9, 2.6]	1.3 [0.8, 1.9]	0.8 [0.4, 2.4]	<0.001
Fatty acid, dicarboxylate								
	Hexadecenedioate (C16:1-DC)	1.6 [1.2, 1.8]	2.1 [1.7, 3.0]	2.3 [1.6, 3.7]	2.3 [1.5, 3.2]	2.3 [1.3, 3.4]	1.6 [1.1, 2.7]	<0.001
	Hexadecanedioate (C16)	1.1 [0.7, 1.6]	1.7 [1.2, 3.0]	1.9 [1.3, 3.6]	2.2 [1.5, 3.4]	2.0 [1.1, 3.4]	1.3 [1.0, 2.7]	<0.001
	Octadecenedioate (C18:1-DC)	1.4 [0.9,1.9]	1.9 [1.3, 2.9]	2.1 [1.3, 3.3]	2.1 [1.6, 2.9]	2.3 [1.2, 2.7]	1.8 [1.2, 3.2	0.03
	Tetradecanedioate (C14)	1.1 [0.7, 1.6]	1.7 [1.1, 2.6]	1.7 [1.1, 2.8]	1.9 [1.1, 2.8]	1.8 [1.3, 2.7]	1.7 [1.0, 2.4]	<0.001
	Octadecanedioate (C18)	1.2 [0.8, 1.8]	1.7 [1.1, 2.7]	1.8 [1.2, 2.9]	2.1 [1.5, 2.9]	1.9 [1.5, 3.2]	1.9 [1.2, 2.4]	0.03
	3-Hydroxydodecanedioate	0.5 [0.1, 0.9]	1.2 [0.5, 2.1]	1.4 [0.7, 2.8]	1.3 [0.7, 2.7]	1.2 [0.7, 2.7]	1.0 [0.4, 1.7]	0.01
	Tetradecadienedioate (C14:2-DC)	1.2 [0.6, 1.7]	1.6 [0.9, 2.6]	1.8 [1.2, 3.0]	1.7 [0.9, 2.7]	1.7 [1.0, 3.0]	1.1 [0.7, 1.8]	<0.001
	3-Hydroxyadipate	0.6 [0.5, 1.0]	1.2 [0.7, 2.3]	1.8 [1.1, 3.0]	1.5 [0.8, 2.3]	1.2 [0.7, 1.7]	1.0 [0.5, 1.6]	0.1

Values for metabolites are shown as median [P25, P75]. FDR comparing metabolites across PH group and controls adjusting for age, sex, race, and eGFR.

In the overall PH cohort, only four known metabolites associate with changes in mitochondrial superoxide production (FDR <0.05). Of these, two are xenobiotics, one is involved in lysine metabolism and the other in tryptophan metabolism. There are no significant associations between mitochondrial superoxide production and metabolites at FDR <0.05 within G1, 2, or 3 PH. No metabolites are significantly associated with mitochondrial mass in the overall PH cohort or within PH groups (all FDR >0.1). Findings suggest that the mitochondrial transmembrane potential across most PH groups is most strongly associated with changes in fatty acid metabolism.

### Mitochondrial function and clinical outcomes

Longitudinal clinical outcomes of death or transplantation of individuals with PH are available for a minimum of 4 years after enrollment to the PVDOMICS study. Mitochondrial measures are not associated with clinical outcomes in the overall PH cohort; however, transmembrane potential is a strong predictor of clinical outcomes of death or transplantation only in G1 PAH, not in the other PH groups. Patients with G1 PAH who die or receive lung transplantation have lower mitochondrial transmembrane potential after adjusting for age, sex, race, and time since diagnosis and the use of PH-specific therapies. The measured transmembrane potential in the 44 individuals who died or had transplantation was 2.8 (2.1, 3.7) mean fluorescence intensity (MFI) × 10^4^ *versus* 3.3 (2.3, 4.7) in the 158 individuals without death/transplant (odds ratio: 0.98 [95% confidence interval: 0.96–1.00]; *p* = 0.04). A survival analysis was performed to test for predictors of death or transplantation using Cox proportional hazards regression models. The variables in the analysis are sex, age, use of PH medications, REVEAL LITE 2.0, and mitochondrial transmembrane potential (or superoxide production or mitochondrial mass). In G1 PAH only, higher transmembrane potential is associated with longer transplant-free survival (Fig. 5). Transmembrane potential is not a predictor of survival in G2 or 3 PH. In addition, superoxide production and mitochondrial mass are not associated with survival in any of the PH groups.

**Figure:**
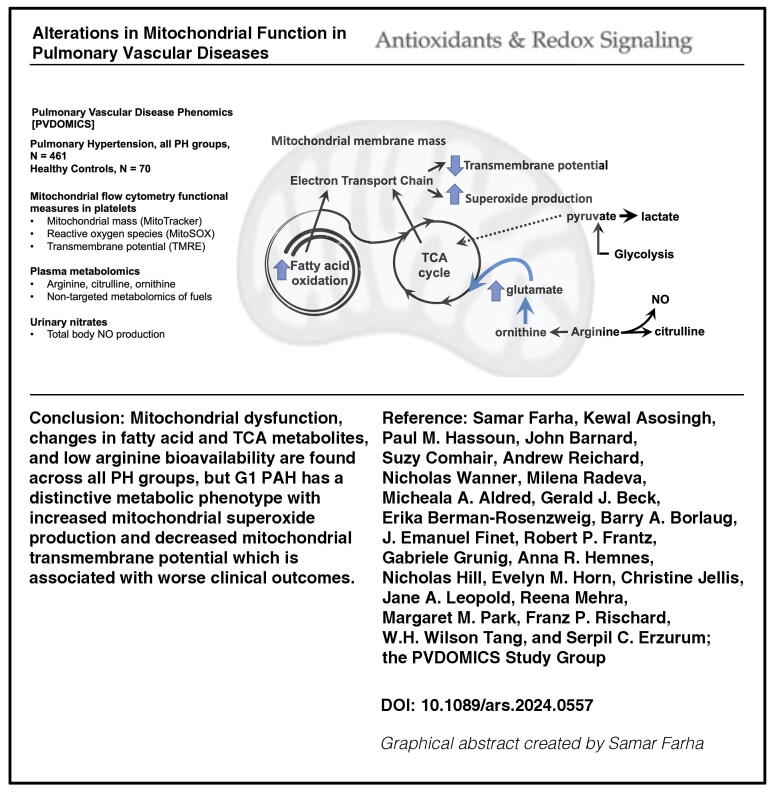


## Discussion

This is the first study measuring mitochondrial function and mass in a large cohort of PH patients and across all five PH groups in comparison with healthy controls. We show alterations in mitochondrial function as well as decreased arginine bioavailability in PH. Decreased arginine bioavailability is found across all PH groups; however, the alterations in mitochondrial function are more characteristic of G1 PAH patients with decreased transmembrane potential compared with controls and other PH groups, associated with higher superoxide production and changes in fatty acid metabolism. Importantly, mitochondrial transmembrane potential is a predictor of transplant-free survival in G1 PAH, with higher levels associated with better clinical outcomes.

Platelet mitochondrial function has been investigated in various medical conditions (Guo et al., 2009; Rezania et al., 2017; Shi et al., 2008). In neurological diseases, specific alterations in ETC activity in platelets are found in Alzheimer’s disease, Huntington’s disease, schizophrenia, migraine headaches, and Parkinson’s disease (Ben-Shachar et al., 2007; Parker et al., 1990a, 1990b; Parker et al., 1989; Sangiorgi et al., 1994; Shi et al., 2008). In PH, there are only three studies that looked at platelet bioenergetics. In two separate studies, Nguyen et al. found that platelets from PAH patients had increased glycolysis and higher maximal uncoupled respiration with increased respiratory reserve capacity compared with control platelets, whereas G2 PH platelets did not have increased glycolysis but demonstrated an increased mitochondrial reserve capacity (Nguyen et al., 2017; Nguyen et al., 2019). Interestingly, there is no difference in platelet basal respiration and oxidant production between PH and controls (Nguyen et al., 2017). The authors showed that the increase in reserve capacity is driven by fatty acid oxidation in PAH platelets. In another report comparing PAH patients with healthy controls, G2 PH patients, and participants who had dyspnea but no PH, platelets from PAH patients have increased basal glycolysis and decreased glycolytic reserve compared with platelets from healthy controls, but no difference in the glycolytic rate compared with G2 PH and/or with patients with dyspnea and no PH (McDowell et al., 2020). Smits et al. analyzed platelet ribonucleic acid (RNA) and showed that platelet RNA signatures distinguish PH patients from controls supporting the use of circulating platelets in early diagnosis of patients with PH and as a disease biomarker (Smits et al., 2022). Altogether, the findings support that platelet bioenergetics is altered in PH and could be used as a disease biomarker. It remains unclear whether platelet mitochondrial function reflects the pulmonary vasculature and right ventricle (RV). Further studies are needed to determine if abnormalities of platelet bioenergetics play a role in the pathogenesis of disease or are a result of the known vascular injury/remodeling and thrombotic disease progression.

The ETC spans the inner mitochondrial membrane and comprises four complexes that work together to transfer electrons from NADH and FADH2 to oxygen (O_2_). During this process, protons are pumped by Complexes I and III into the intermembrane space creating a proton gradient that powers Adenosine Triphosphate (ATP) synthesis *via* Complex V. The TCA cycle and fatty acid oxidation are essential sources for NADH and FADH2. Thus, mitochondrial ETC activity is closely linked to fatty acid oxidation and TCA cycle. All must cooperate to provide the energy needed for cell functions. In this study, while there are no associations between transmembrane potential and TCA pathway metabolites, there are differences in TCA metabolites among PH and controls and a much higher lactate-to-pyruvate ratio in G1 PAH. This confirms a shift to glycolysis, with less pyruvate oxidation in PAH (Xu et al., 2021). In contrast, transmembrane potential is associated with fatty acid pathways, that is, fatty acyl carnitine dicarboxylate metabolism. Fatty acyl carnitine dicarboxylate metabolism enables transport and processing of long-chain fatty acids into the mitochondria for oxidation. Inside the mitochondria, fatty acyl carnitine is converted to fatty acyl CoA, which is broken down through β-oxidation for the generation of NADH and FADH2. The findings in this study suggest that G1 PAH may have increased reliance on fatty acid oxidation for energy, and preference for entry of reducing equivalents directly into Complex III (Gnaiger, 2024).

Imbalance in different types of ROS is well described in PH and linked to pathogenesis (Breault et al., 2023; Sutendra and Michelakis, 2014). Previous studies measured hydrogen peroxide in the pulmonary vasculature of hypoxic and nonhypoxic PH models and found different results based on experimental conditions, species, and hypoxic conditions emphasizing the complexity of mitochondrial ROS changes related to disease stage, etiology, species/model, and tissue studied (Afolayan et al., 2016; Archer et al., 2010; Archer et al., 2008; Bonnet et al., 2006; Chi et al., 2010; Nozik-Grayck et al., 2008; Waypa et al., 2010). Mitochondrial number or mass, which controls cellular bioenergetic capacity, was shown to be reduced in the skeletal muscle of PH patients and in the pulmonary vasculature and RV of rat models of PH (Archer et al., 2008; Bonnet et al., 2006; Nisoli and Carruba, 2006; Xu et al., 2007). Nguyen et al. found no difference in mitochondrial superoxide production in platelets from G1 PAH, G2 PH, and controls (Nguyen et al., 2017; Nguyen et al., 2019). In this study, G1 PAH has higher superoxide production compared with G3 PH and controls. This suggests that the metabolic mechanisms underlying PAH differ from those of G3 PH.

The study has limitations. One important limitation is the difficulty in accounting for the effect of disease duration and PH therapies on platelet mitochondrial function and more so the specific therapeutic subclasses. Studies investigating platelet mitochondrial function over time and in response to therapy are warranted to help understand the effect of PH therapies and disease progression on platelets. Based on our findings, it is difficult to determine if the changes in platelet mitochondrial function are a cause or an effect of PH. Nonetheless, the differences in mitochondrial function among the PH groups suggest that it is linked to disease pathogenic mechanisms. Extrapolating from studies in heart failure (Rosca and Hoppel, 2010), mitochondrial dysfunction could be both the cause and the result of disease pathogenesis leading to metabolic reprogramming, increased oxidative stress, and RV failure. Another limitation is the lack of platelet functional mechanistic assays, which were beyond the scope of the current study. G4 and 5 PH was underrepresented in our cohort and a larger sample size is needed to better characterize mitochondrial mass and function in these two groups. Another important modulator of mitochondrial function that was not accounted for in this study is the loss of bone morphogenetic protein receptor type II function and its association with mitochondrial dysfunction and oxidative stress in PH (Cuthbertson et al., 2023; Diebold et al., 2015; Egnatchik et al., 2017; Lane et al., 2011).

In summary, decreased arginine bioavailability is found in all PH groups; whereas mitochondrial functions vary among PH groups. Lower transmembrane potential with increased superoxide production is a unique characteristic of G1 PAH and is associated with changes in fatty acid metabolism. The transmembrane potential is predictive of clinical outcomes in G1 PAH. The G1 PAH findings are in contrast to G3, where superoxide is lower and transmembrane potential is higher, suggesting different metabolic states among these groups. Further studies are needed to investigate mitochondrial mechanisms in all PH and to identify targets for specific metabolic interventions to improve clinical outcomes.

## Materials and Methods

### Study population and sample collection

The PVDOMICS study is an observational prospective longitudinal cohort (clinicaltrials.gov NCT02980887). The study protocol and a STROBE diagram depicting enrollment and patient classification have been previously published (Hemnes et al., 2022). The study was approved by the Institutional Review Board of the Cleveland Clinic and all clinical sites enrolling participants. It was sponsored by the National Institutes of Health Heart, Lung and Blood Institute and the Pulmonary Hypertension Association. Written informed consent was obtained from all individuals. Incident and prevalent patients with PH, disease comparators, and age-, sex-, race-, and Hispanic ethnicity-matched healthy controls were recruited from seven clinical sites across the United States (Hemnes et al., 2022). The present study involved two groups: patients with PH according to the Fifth World Symposium on Pulmonary Hypertension guidelines with mean pulmonary arterial pressure (mPAP) ≥25 mmHg and healthy control subjects with normal cardiopulmonary findings and without end-organ disease. All participants were ≥18 years old. The inclusion criteria for patients with PH were the following: age ≥18 years, referred for right heart catheterization for clinical purposes, able to perform complete diagnostic testing, treatment naive, or prior drug exposure. Exclusion criteria were end-stage renal disease requiring renal replacement therapy, too ill to perform study protocol, pregnant or nursing, and active malignancy other than nonlocalized skin cancer. Following right heart catheterization, PH participants were classified according to the 2013 World Symposium on Pulmonary Hypertension guidelines that were in effect at the inception of the study (Simonneau et al., 2013). PH was defined by right heart catheterization, as mPAP ≥25 mmHg, and PAH was defined as mPAP ≥25 mmHg, and pulmonary vascular resistance >3 Wood Units with pulmonary artery wedge pressure ≤15 mmHg. Patients were assigned PH groups based on the five recognized groups of disorders that cause PH: PAH (G1); PH due to left heart disease (G2); PH due to chronic lung disease and/or hypoxia (G3); chronic thromboembolic PH (G4); and PH due to unclear multifactorial mechanisms (G5). Subjects were classified as single or mixed etiology PH at the discretion of the site principal investigator. For this study, analysis was performed using the single etiology PH group unless otherwise specified. Participants were assigned to appropriate groups by investigators with the oversight of an Adjudication Committee. Healthy controls underwent all noninvasive testing and blood sampling but did not undergo right heart catheterization or ventilation/perfusion scanning. Comprehensive clinical phenotyping was performed through standardized cardiopulmonary testing, including pulmonary function testing, 6-minute walk distance (6MWD) testing, echocardiography, cardiac magnetic resonance imaging, resting right heart catheterization with O_2_, and vasodilator challenge followed by fluid challenge or invasive cardiopulmonary exercise testing and measurement of N-terminal pro-brain natriuretic peptide (NT-proBNP) as previously described (Hemnes et al., 2022). REVEAL LITE 2.0 risk was calculated by incorporating NT-proBNP, 6MWD, the World Health Organization functional class, systolic blood pressure, heart rate, and estimated glomerular filtration rate (eGFR) (Benza et al., 2021). Peripheral venous blood was collected in BD Vacutainer 4 mL sodium–heparin blood tubes (BD, Cat# 367871, San Jose, CA) and processed the following morning after overnight shipment to the Cleveland Clinic from the other study institutions. Samples were not stored. Hemolyzed samples were excluded and a total of 767 samples were processed. All samples were deidentified. Electronic laboratory notebook was used.

### Measurement of mitochondrial function and mass by flow cytometry

#### Mouse splenocyte reference sample preparation

Mouse splenocyte reference samples were used daily to help ensure that flow cytometry data acquisition each day was not impacted by flow cytometer setup, sample preparation, staining, or any other variables. Acceptable reference ranges for MFI of each mitochondrial probe were calculated following three consecutive days of data acquisition using batches of isolated mouse splenocytes frozen in liquid nitrogen, with average MFI ±2 standard deviation (SD) determining the reference range. Mouse splenocyte reference samples were stained and run daily with the test platelet samples.

#### Isolation and processing of platelets from whole blood

Blood tubes were inverted to gently mix following shipment and centrifuged at 150 *g* for 20 min with the brake turned off. The yellow layer of platelet-rich plasma was carefully collected from each tube using a 1 mL micropipette and transferred into a 15 mL polyethylene tube. For each milliliter of platelet-rich plasma collected, 1 µL of 10 mM prostaglandin E1 (PE1) was added. PE1 is used to inhibit platelet aggregation (Xu et al., 2015). Platelet-rich plasma was centrifuged at 150 *g* for 10 min to avoid red blood cell contamination that may have been inadvertently included in the platelet-rich plasma following the initial centrifugation. The supernatant was transferred into a new 15 mL polyethylene tube. This suspension was centrifuged at 1500 *g* for 15 min with the acceleration and brake both set to maximum. Platelet pellets were resuspended in 1 mL of Tyrode’s buffer/PE1. Platelets were counted using a TC20 Cell Counter (Bio-Rad, Cat#145–0102, Hercules, CA).

#### Mitochondrial function flow cytometry assay

Platelet pellets in four of the five mini flow tubes were resuspended in 90 µL of Tyrode’s buffer/PE1. The platelet pellet in the fifth tube was resuspended in 90 µL of 20 µM FCCP (carbonyl cyanide *p*-trifluoromethoxyphenylhydrazone) in Tyrode’s buffer/PE1 and incubated for 10 min. Each of the five tubes then received an additional 10 µL of specific MitoProbe dilutions to reach a final volume of 100 µL. All MitoProbe had a stock concentration of 1 mM. One tube was an unstained control and therefore only received an additional 10 µL of Tyrode’s buffer/PE1. One tube resuspended in Tyrode’s buffer/PE1 and the tube treated with FCCP/Tyrode’s buffer/PE1 received 10 µL of a 1/250 dilution of TMRE (tetramethyl rhodamine, ethyl ester, Abcam, Cat#113852, Cambridge, UK) for a final dilution of 1/2500 TMRE. One tube received 10 µL of a 1/100 dilution of MitoTracker Green (Invitrogen, Cat#M7514, Waltham, MA) for a final dilution of 1/1000 MitoTracker Green. The final tube received 10 µL of a 1/25 dilution of MitoSOX Red (Invitrogen, Cat#M36008, Waltham, MA) for a final dilution of 1/250. Resuspend platelets and incubate in the dark for 30 min at 37°C. Following incubation, 1 mL of Tyrode’s buffer/PE1 was added to the four tubes without FCCP and 1 mL of FCCP/Tyrode’s buffer/PE1 for the tube that previously received FCCP. All tubes were centrifuged at 3100 *g* for 4 min and platelet pellets were resuspended in 250 µL of Tyrode’s buffer/PE1 (250 µL FCCP/Tyrode’s buffer/PE1 for FCCP tube). Samples were stored in the dark with the unstained, MitoTracker Green, and MitoSOX Red samples at 4°C and the TMRE and TMRE/FCCP samples at 37°C until data acquisition.

#### Data acquisition on flow cytometer

Data acquisition was performed on a BD LSR Fortessa Flow Cytometer (BD, San Jose, CA) using a blue laser (Ar488) with a 515/20 filter for MitoTracker Green, blue laser (Ar488) with an LP535 dichroic and 585/42 filter for MitoSOX Red, and a yellow green laser (561) with 582/15 filter for TMRE. The six peak SHERO Ultra Rainbow Calibration Kit (Spherotech, Cat#URCP-50-2K, Lake Forest, IL) was used to standardize fluorescence detectors. A total of 50,000 events were acquired for all platelet tubes and 10,000 events were acquired for all mouse splenocyte reference sample tubes. Data were saved as Flow Cytometry Standard (FCS) files on the institutional server.

### Data analysis

Data analysis was performed using FlowJo X software by investigators blinded to the diagnosis associated with the samples. All raw data acquired for each sample were displayed on forward scatter or FSC-A × FSC-H light scatter and gated tightly along the diagonal axis for aggregate exclusion. This population was then displayed on side scatter or SSC-A × SSC-H light scatter and gated tightly along the diagonal axis for debris exclusion. These gated single platelet events were then evaluated for fluorescence, and MFI was calculated in the channel corresponding to each probe (Data acquisition on flow cytometer), using the following gating strategies (Supplementary Fig. S1).

For MitoTracker Green-stained samples, no further gating was used and all single platelet events were used to calculate MFI in Blue-515, measuring the mitochondrial mass present in each individual sample. Unstained single platelet MFI in Blue-515 was subtracted from the MFI of the MitoTracker Green-stained single platelets for background correction. Thirty-one samples showed significant populations with negative fluorescence in Blue-515 and were excluded due to this abnormal fluorescent signal. Fourteen samples had single platelet event counts <5000 and were excluded due to insufficient events for accurate flow cytometric analysis.

For MitoSOX Red-stained samples, MitoSOX Red-positive events were gated in Blue-710 and the MFI of this population was calculated to measure superoxide presence within the platelets. Unstained single platelet MFI in Blue-710 was subtracted from the MFI of the MitoSOX Red-stained single platelets for background correction. Four samples had distorted light scatter inconsistent with MitoSOX Red fluorescent signals indicating sample degradation and were excluded. One sample had only 1000 total single platelet events collected and was excluded. One sample was contaminated with TMRE and was excluded.

For TMRE-stained samples, TMRE-positive events were gated in YG-582 and the MFI of this population was calculated, measuring mitochondrial transmembrane potential in the samples. MFI in YG-582 of all single platelet events from the TMRE+FCCP sample was subtracted from the MFI of the TMRE-stained single platelets for background correction. One sample had no TMRE signal measured due to possible MitoSOX Red contamination and was excluded. One sample had an abnormal signal in the TMRE+FCCP tube used for background correction and the sample was excluded. One sample had no TMRE data collected and was therefore excluded.

In total, 767 samples were collected and prepared for data acquisition on the flow cytometer. Forty-three samples had MitoTracker Green data excluded, leaving a total of 724 data points. Six samples had MitoSOX Red data excluded, leaving a total of 761 data points. Three samples had TMRE data excluded, leaving a total of 764 data points. A total of 717 samples have data points for all three stains. Background-adjusted MFIs for all nonexcluded samples were reported.

Mitochondrial probe MFI of mouse splenocyte reference samples had to fall within the established reference range for the platelet data to be included in our final analysis.

### Arginine–NO metabolism

#### Plasma amino acid and urinary nitrate analysis

Plasma arginine, citrulline, and ornithine concentrations were measured using high-performance liquid chromatography (HPLC; Agilent 1100 series HPLC; Agilent Technologies; Wilmington, DE), following ortho-phthalaldehyde derivatization using a fluorescent detector as described previously (Kalhan et al., 2003).

Nitrate in urine samples was measured to assess whole-body NO production (Castillo et al., 1993). Urine nitrate was measured using the ENO-30, a dedicated HPLC system (Amuza, San Diego, CA). Urine was diluted 1:50 in the analyzer carrier solution before running. Urine samples were also screened for the presence of nitrite (*i.e.,* urinary tract microbial infection) with the Griess reaction, and samples testing positive for nitrite were excluded from analysis. Urine creatinine was measured in all samples, and nitrate concentration was normalized to creatinine concentration.

#### Nontargeted metabolomic analysis

Nontargeted metabolomic and lipidomic analyses were performed at Metabolon, Inc. (Durham, NC). The details of analytical platform and data curation have been described in detail previously (Comhair et al., 2015). The global, unbiased platform was based on a combination of three separate platforms: ultrahigh performance liquid chromatography/tandem mass spectrometry (UHPLC/MS/MS) optimized for basic species, UHPLC/MS/MS optimized for acidic species, and gas chromatography/mass spectrometry (GC/MS). The major components of the analytic process and the analytic platform have been described in detail in previous publications (Comhair et al., 2015; Evans et al., 2009; Kalhan et al., 2011). UHPLC/MS/MS analysis utilized a Waters Acquity UHPLC (Waters Corporation, Milford, MA) coupled to an Linear Trap Quadrupole (LTQ) mass spectrometer (Thermo Fisher Scientific, Inc., Waltham, MA) equipped with an electrospray ionization source. Two separate injections were performed on each sample: one optimized for positive ions and one for negative ions. Derivatized samples for GC/MS were analyzed on a Thermo-Finnigan Trace DSQ fast-scanning single-quadrupole MS operated at unit mass resolving power. Chromatographic separation followed by full-scan mass spectra was carried to record retention time, mass-to-charge (m/z) ratio, and MS/MS of all detectable ions present in the samples. Compounds were identified by automated comparison with Metabolon’s reference library entries. Identification of known chemical entities was based on comparison with Metabolon’s library entries of purified standards.

### Statistical analysis

Quantitative variables were summarized with means and SDs or medians and interquartile ranges as appropriate depending on skewness, while categorical variables were summarized as frequencies and percents. Group comparisons were done using Pearson’s chi-square or Fisher’s exact test for categorical variables. We used analysis of variance or Kruskal–Wallis for continuous variables at 0.05 significance levels, with pairwise group comparisons performed with two-tailed *t*-tests or Wilcoxon test as appropriate at Bonferroni-adjusted significance levels to account for multiple comparisons. Pearson’s correlations were used to assess the relationships between continuous variables and a line of best fit used in figures for visualization. Multivariable regression models were used to test the differences in mitochondrial measures comparing healthy controls and PH adjusting for age, sex, and race. To adjust for PH medications, propensity scores were calculated using the Inverse Probability Treatment Weighting (IPTW) method. Sex, age, and race were used as confounders. Generalized linear models adjusted for PH medications were used to test the differences of the mitochondrial measures between the PH groups. Bonferroni-adjusted significance level <0.005 for multiple comparisons were reported. Analyses were performed on the data as of January 30, 2023, using JMP Pro, version 17.0 (SAS Institute) and SAS software (version 9.4, Cary, NC).

## Statistical Analysis of Untargeted Metabolomics

### Filtering and scaling

Baseline venous plasma untargeted metabolomic data from Metabolon were filtered per metabolite using all PVDOMICS participants so that the remaining metabolites had <50% missing values and their ratio of median absolute deviation (MAD)-to-median value was ≥0.25. Each metabolite was rescaled to have an MAD and median of 1 using all PVDOMICS participants. Filtered and scaled metabolite data were then subsetted to those with PH or healthy controls and complete platelet mitochondrial mass and mitochondrial function data.

### Imputation of clinical covariates

Missing data in key clinical covariates, eGFR (46 missing), race (13 missing), PH medication use (3 missing), and body mass index (BMI) (2 missing) were imputed using Random Forest imputation (Stekhoven and Buhlmann, 2012) and additional complete predictors recruiting center, gender, age, PH status, PH group, mitomass, and mitochondrial function, and 50 principal components from the scaled, filtered, and subsetted metabolite data after log2 transformation.

### Differential metabolite abundance

Differential metabolite abundance analyses were performed per metabolite using a robust and efficient semiparametric regression modeling approach (Liu et al., 2017) with a log–log link. Additive regression models per metabolite (outcome) were fit using maximum likelihood and the PH-only subset (all PH groups) with covariate adjustment for recruiting center, gender, age, BMI, eGFR, race, PH medication use, PH group, and platelet mitochondrial mass, transmembrane potential, or superoxide production. Age, BMI, eGFR, and mitochondrial variables were included as 2 degree-of-freedom restricted cubic splines to allow for nonlinear effects. *p* Values per mitochondrial predictor were calculated using likelihood-ratio tests, then adjusted per predictor to get FDR-controlled values using the Benjamini–Hochberg method (Benjamini and Hochberg, 1995). Effect sizes of mitochondrial predictors on metabolites were calculated by assessing the change in predicted median metabolite values as the mitochondrial predictor changed (typically from its first to third quartile), while all other predictors in the model remained constant at a specified value (typically at their modal or median values).

To assess interaction effects of PH group on the metabolite–mitochondrial measure relationships, the regression models were refit to include a PH group by mitochondrial measure interaction term (both linear and nonlinear) but restricted to PH groups 1–3 due to small sample sizes in PH groups 4 and 5. Overall *p* values of interaction strength were calculated using likelihood ratio tests, and contrasts of metabolite–mitochondrial relationships in PH 2 or 3 *versus* PH 1 were estimated. Finally, PH1–3-specific models were fit.

Metabolite pathway analyses were conducted for each of the models using the corresponding individual metabolite–mitochondrial association *p* values as inputs, the Metabolon-provided pathway annotations, and the principled R-package Simultaneous Enrichment Analysis (rSEA) method (Ebrahimpoor et al., 2020). The competitive null hypothesis of a higher true discovery proportion (TDP) for metabolites in the pathway *versus* outside the pathway was tested for every pathway. The rSEA method controlled the overall family-wise error rate across all possible pathways from the tested metabolites. The estimated TDP was reported for each pathway.

## Survival Analysis of Platelet Mitochondrial Measures

Imputation of clinical covariates and survival analyses were done separately within each of the PH groups 1–3. Survival event was composite of death or transplant.

### Imputation of clinical covariates

Missing data in clinical covariates defining the REVEAL LITE 2 risk score and key covariates from the metabolite models above were imputed using Random Forest imputation (Stekhoven and Buhlmann, 2012) and additional complete predictors recruiting center, gender, age, PH status, World Symposium on Pulmonary Hypertension (WSPH) group, mitomass and mitochondrial function, death or transplant status, time to death or transplant or last follow-up, and number of years since PH diagnosis.

### Survival analysis

Cox proportional hazards models were fit for the composite death or transplant event with noninformative censoring at time of last follow-up applied to those without an event. Baseline hazards were stratified by PH medication use. Covariate model included gender, age, REVEAL LITE 2 score, and platelet mitochondrial mass, transmembrane potential, or superoxide production. Age, REVEAL LITE 2 score, and mitochondrial variables were included as 2 degree-of-freedom restricted cubic splines to allow for nonlinear effects. *p* Values per mitochondrial predictor were calculated using likelihood-ratio tests. Effects of mitochondrial predictors on survival were displayed as plots of predicted 4-year survival probabilities as the mitochondrial predictor changed (typically from its first to third quartile), while all other predictors in the model remained constant at a specified value (typically at their modal or median values). Proportional hazard assumptions were checked and tested using Schoenfeld residual plots and tests.

All metabolite and survival analyses were conducted using R version 4.3 and the R packages survival, data.table, rms, and Hmisc.

## Abbreviations Used

6MWD: 6-minute walk distance
ATP: Adenosine Triphosphate
BMI: body mass index
eGFR: estimated glomerular filtration rate
ETC: electron transport chain
FADH2: Flavin Adenine Dinucleotide
FCCP: carbonyl cyanide *p*-trifluoromethoxyphenylhydrazone
FCS: Flow Cytometry Standard
FDR: false discovery rate
FSC: forward scatter
G: group
GABR: global arginine bioavailability ratio
GC/MS: gas chromatography/mass spectrometry
HPLC: high-performance liquid chromatography
IPTW: Inverse Probability Treatment Weighting
LTQ: Linear Trap Quadrupole
MAD: median absolute deviation
MFI: mean fluorescence intensity
mPAP: mean pulmonary arterial pressure
NADH: Nicotinamide Adenine Dinucleotide
NO: nitric oxide
NT-proBNP: N-terminal pro-brain natriuretic peptide
O_2_: oxygen
PAH: pulmonary arterial hypertension
PE1: prostaglandin E1
PH: pulmonary hypertension
PVDOMICS: Pulmonary Vascular Disease Phenomics
RNA: ribonucleic acid
ROS: reactive oxygen species
rSEA: R-package Simultaneous EnrichmentAnalysis
RV: right ventricle
SD: standard deviation
SEM: Standard Error of the Mean
SSC: side scatter
TCA cycle: tricarboxylic acid cycle
TDP: true discovery proportion
TMRE: tetramethyl rhodamine
UHPLC/MS/MS: ultrahigh-performance liquid chromatography/tandem mass spectrometry
WSPH: World Symposium on Pulmonary Hypertension
