# Supplementary material for: Alterations in Mitochondrial Function in Pulmonary Vascular Diseases
Source: Antioxid Redox Signal. 2025 Mar 7;42(7-9):361–77. doi: 10.1089/ars.2024.0557 (PMC12344126; doi:10.1089/ars.2024.0557)

Supplemental Figure 1. Events were plotted on a forward scatter (FSC) and side scatter (SSC) plots on log scale and singlets were gated as shown (upper panel). Control staining for mitoTracker and mitoSOX included samples stained with buffer without probe. Control staining for TMRE included the same sample treated with FCCP to collapse mitochondrial potential. Overlays of controls and probes are shown (lower panel).

Gating Strategy – Light Scatter. All Samples were gated on FSC-A x FSC-H followed by SSC-A x SSC-H for aggregate exclusion.

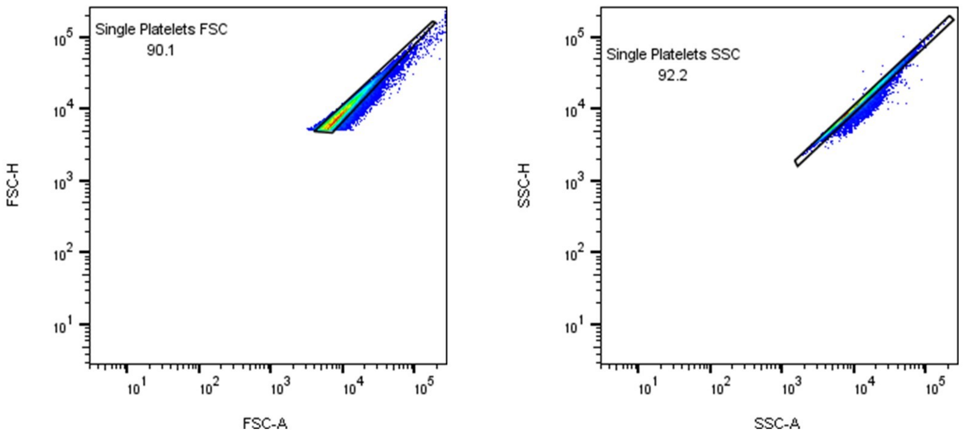

Overlays of control (unstained or FCCP treated) samples and mitochondrial probes.

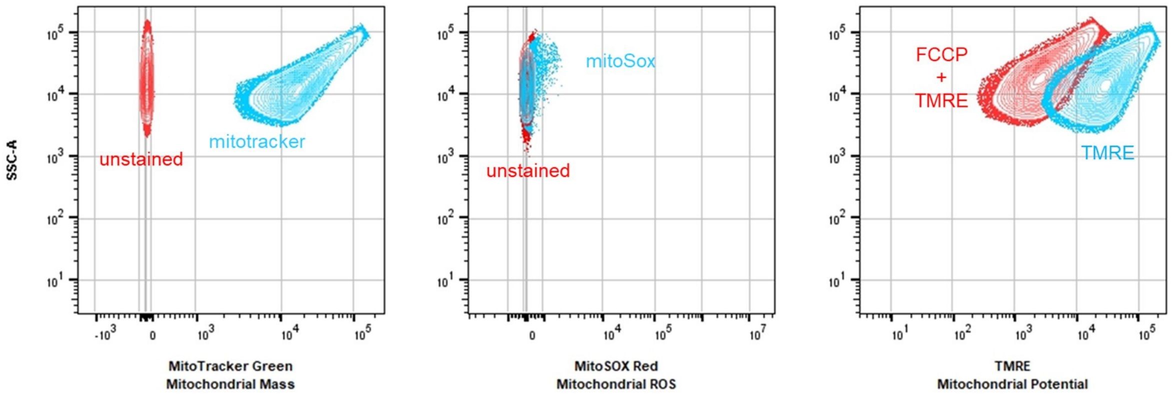

Supplement: Supplementary Figure S1 [file ars.2024.0557_supp_figs1.pdf]
