# Supplementary material for: Alterations in Mitochondrial Function in Pulmonary Vascular Diseases
Source: Antioxid Redox Signal. 2025 Mar 7;42(7-9):361–77. doi: 10.1089/ars.2024.0557 (PMC12344126; doi:10.1089/ars.2024.0557)
Supplement: Supplementary Table S1 [file ars.2024.0557_supp_tables1.docx]

**Supplement Table 1. Propensity score analysis using inverse probability of treatment weighing (IPTW method) to adjust for PH therapy**

|  | **G1 PH** | **G2 PH** | **G3 PH** | **G4 PH** | **G5 PH** | **p-value** |
| --- | --- | --- | --- | --- | --- | --- |
| Mitochondrial Mass (MFI) | 1.64  (1.52, 1.76) | 1.7  (1.55, 1.86) | 1.62  (1.49, 1.75) | 1.66  (1.43, 1.88) | 1.83  (1.51, 2.15) | 0.65 |
| Mitochondrial Superoxide production (MFI x 10^2^) | 7.1  (6.75, 7.45) ^*^ | 6.86  (6.39, 7.33) | 6.1  (5.7, 6.5) ^*^ | 7.04  (6.38, 7.7) | 6.24  (5.27, 7.22) | <0.01 |
| Mitochondrial Transmembrane potential (MFI x 10^4^) | 3.55  (3.26, 3.84) | 3.94  (3.54, 4.33) | 3.97  (3.64, 4.3) | 3.87  (3.32, 4.43) | 3.84  (3.02, 4.67) | <0.01 |
| Arginine (µM) | 104.5  (99.49, 109.51) | 97.43  (90.62, 104.24) | 102.23  (96.53, 107.93) | 106  (96.48, 115.51) | 100.43  (86.82, 114.04) | 0.65 |
| Ornithine (µM) | 75.77  (71.47, 80.07) ^*^ | 90.7  (84.85, 96.55) ^*†^ | 76.73  (71.84, 81.63) ^†^ | 77.79  (69.63, 85.96) | 74.16  (62.48, 85.85) | <0.01 |
| Citrulline (µM) | 41.16  (38.62, 43.69) ^*^ | 49.17  (45.72, 52.61) ^*†^ | 38.44  (35.56, 41.33) ^†^^ | 47.23  (42.41, 52.04) ^^^ | 43.73  (36.85, 50.62) | <0.01 |
| GABR | 0.93  (0.89, 0.98) ^*^ | 0.76  (0.7, 0.82) ^*†^ | 0.94  (0.89, 0.99) ^†^ | 0.88  (0.8, 0.97) | 0.88  (0.76, 1.01) | <0.01 |
| Urinary nitrate/creatinine  (μmol/μmol) | 0.07  (0.06, 0.08) | 0.06  (0.04, 0.08) | 0.1  (0.08, 0.11) | 0.07  (0.04, 0.09) | 0.06  (0.01, 0.1) | 0.05 |

*Statistics presented as least square mean and 95% CI. GABR: global arginine bioavailability ratio, MFI: mean fluorescence intensity. Sex, age, and race were used as confounders. Generalized linear models adjusted for PH meds were used to test the differences of the mitochondrial measures between the PH groups. Significant pairwise analyses are marked with *, †, ^. Bonferroni-adjusted significance level <0.005 was used for multiple comparisons.*
